# Predictors of viral suppression and rebound among HIV-positive men who have sex with men in a large multi-site Canadian cohort

**DOI:** 10.1186/s12879-016-1926-z

**Published:** 2016-10-21

**Authors:** Zachary Tanner, Nathan Lachowsky, Erin Ding, Hasina Samji, Mark Hull, Angela Cescon, Sophie Patterson, Jason Chia, Alia Leslie, Janet Raboud, Mona Loutfy, Curtis Cooper, Marina Klein, Nima Machouf, Christos Tsoukas, Julio Montaner, Robert S. Hogg, for the Canadian Observation Cohort (CANOC) Collaboration, Robert Hogg, Ann N. Burchell, Curtis Cooper, Deborah Kelly, Marina Klein, Mona Loutfy, Nima Machouf, Julio Montaner, Janet Raboud, Chris Tsoukas, Stephen Sanche, Alexander Wong, Tony Antoniou, Ahmed Bayoumi, Mark Hull, Bohdan Nosyk, Angela Cescon, Charlie Goldsmith, Silvia Guillemi, P. Richard Harrigan, Marianne Harris, Sean Hosein, Sharon Johnston, Claire Kendall, Clare Liddy, Viviane Lima, David Marsh, David Moore, Alexis Palmer, Sophie Patterson, Peter Phillips, Anita Rachlis, Sean B. Rourke, Hasina Samji, Marek Smieja, Benoit Trottier, Mark Wainberg, Sharon Walmsley, Chris Archibald, Ken Clement, Fred Crouzat, Monique Doolittle-Romas, Laurie Edmiston, Sandra Gardner, Brian Huskins, Jerry Lawless, Douglas Lee, Renee Masching, Stephen Tattle, Alireza Zahirieh, Claire Allen, Guillaume Colley, Jason Chia, Daniel Corsi, Louise Gilbert, Alia Leslie, Lucia Light, David Mackie, Costas Pexos, Susan Shurgold, Leah Szadkowski, Chrissi Galanakis, Ina Sandler, Benita Yip, Jaime Younger, Julia Zhu

**Affiliations:** 1 BC Centre for Excellence in HIV/AIDS, Vancouver, Canada; 2 School of Public Health & Social Policy, University of Victoria, Victoria, Canada; 3 Centre for Addiction Research British Columbia, University of Victoria, Victoria, Canada; 4 Faculty of Medicine, University of British Columbia, Vancouver, Canada; 5 Northern Ontario School of Medicine, Sudbury, Canada; 6 Faculty of Health Sciences, Simon Fraser University, Vancouver, Canada; 7 Dalla Lana School of Public Health, University of Toronto, Toronto, Canada; 8 Toronto General Research Institute, University Health Network, Toronto, Canada; 9 Faculty of Medicine, University of Toronto, Toronto, Canada; 10 Maple Leaf Medical Clinic, Toronto, Canada; 11 Women’s College Research Institute, Women’s College Hospital, Toronto, Canada; 12 The Ottawa Hospital Research Institute, University of Ottawa, Ottawa, Canada; 13 Faculty of Medicine, McGill University, Montreal, Canada; 14 The Montreal Chest Institute, McGill University Health Centre, Montreal, Canada; 15 Clinique Medicale l’Actuel, Montreal, Canada; 16 Faculty of Health Sciences, Simon Fraser University, BLU 9512, 8888 University Drive, Burnaby, BC V5A 1S6 Canada

**Keywords:** Canada, HIV, MSM, Viral load, Suppression, Rebound

## Abstract

**Background:**

Gay, bisexual and other men who have sex with men (MSM) are disproportionately affected by HIV in Canada. Combination antiretroviral therapy has been shown to dramatically decrease progression to AIDS, premature death and HIV transmission. However, there are no comprehensive data regarding combination antiretroviral therapy outcomes among this population. We sought to identify socio-demographic and clinical correlates of viral suppression and rebound.

**Methods:**

Our analysis included MSM participants in the Canadian Observational Cohort, a multi-site cohort of HIV-positive adults from Canada’s three most populous provinces, aged ≥18 years who first initiated combination antiretroviral therapy between 2000 and 2011. We used accelerated failure time models to identify factors predicting time to suppression (2 measures <50 copies/mL ≥30 days apart) and subsequent rebound (2 measures >200 copies/mL ≥30 days apart).

**Results:**

Of 2,858 participants, 2,448 (86 %) achieved viral suppression in a median time of 5 months (Q1–Q3: 3–7 months). Viral suppression was significantly associated with later calendar year of antiretroviral therapy initiation, no history of injection drug use, lower baseline viral load, being on an initial regimen consisting of non-nucleoside reverse-transcriptase inhibitors, and older age. Among those who suppressed, 295 (12 %) experienced viral rebound. This was associated with earlier calendar year of antiretroviral therapy initiation, injection drug use history, younger age, higher baseline CD4 cell count, and living in British Columbia.

**Conclusions:**

Further strategies are required to optimize combination antiretroviral therapy outcomes in men who have sex with men in Canada, specifically targeting younger MSM and those with a history of injection drug use.

## Background

Gay, bisexual and other men who have sex with men (MSM) have the highest prevalence of HIV in Canada [1]. Between 1985 and 2011, 54.7 % of diagnosed HIV cases with known exposure status in Canada were attributable to MSM (*n* = 69,856), even though self-identified MSM comprise only an estimated 2.1 % of the Canadian population [1, 2]. In the first two decades of the epidemic, this disproportionate burden was characterized by premature mortality across MSM communities [3], with the estimated life expectancy of gay men in some urban environments being 8 to 20 years less than that of the general male population [4]. Since the implementation of combination antiretroviral therapy (cART), people living with HIV/AIDS (PHAs) have experienced significant improvements in health outcomes and can now achieve life expectancy near that of the general population [5–7]. High levels of adherence to cART, usually defined as taking >95 % of prescribed medication [8], usually results in full suppression of HIV-1-RNA levels in plasma, markedly improving health outcomes and similarly reducing the risk of HIV transmission [9]. Adherent patients generally achieve viral suppression between 8 and 24 weeks after initiating treatment [10].

Despite the proven clinical benefits of cART, Canadian MSM continue to experience a sustained rate of new HIV infections compared with the general population [1, 11]. A myriad of factors, including the heightened risk of infection via anal sex compared with vaginal sex [12]; behavioural factors, such as condomless anal intercourse and substance use [13]; and social factors, including homophobia, stigma, and social exclusion [11, 12, 14] have been advanced to explain these high rates of new infections. Additionally, structural barriers such as criminalization of HIV exposure [15], insufficient access to culturally appropriate health services and distrust of available health care providers [16–18], may deter some MSM from seeking HIV testing and treatment, as well as negatively impact retention rates. Continuation of treatment is crucial for long-term clinical success and prevention of viral rebound [19]. Failure to remain virally suppressed increases the risk of poor health outcomes, such as HIV drug resistance, progression to AIDS, premature death, and HIV transmission [20, 21].

While risk factors for HIV seroconversion among MSM have been largely explored, there is a sizable research gap regarding modern-day cART treatment responses. To date, there has not been an analysis of the clinical and social circumstances associated with virologic outcomes among MSM in Canada. The Public Health Agency of Canada has specifically identified this knowledge gap [1]. Furthermore, viral suppression is a principal component of the new UN 90-90-90 Target (i.e., to achieve 90 % of PHA diagnosed globally, 90 % of them on treatment, 90 % of them on cART virally suppressed by 2020) [22]. This ambitious plan to end AIDS as a global pandemic provides a timely and pertinent framework for assessing where Canadian MSM on cART stand with regard to this target. The purpose of this study was to identify socio-demographic and clinical correlates of treatment response among MSM in Canada, as measured by viral suppression and subsequent virologic rebound. This will help inform cART retention strategies for MSM living with HIV.

## Methods

### Study population

The Canadian Observational Cohort (CANOC) collaboration is an observational cohort study of antiretroviral-naïve HIV-positive individuals initiating cART after 1 January 2000 [23]. This multi-site study is comprised of eight cohorts located in BC, Quebec, and Ontario. Almost half of the estimated 20,500 HIV-positive individuals on cART in these three provinces are represented within CANOC [24]. Patient eligibility criteria for inclusion in CANOC are: documented HIV infection, residence in Canada, aged 18 years or older, initiation of a first antiretroviral regimen comprised of at least three individual agents, and at least one measurement of HIV plasma viral load and CD4 T-cell count within one year of initiating cART. Patient selection and data extraction are performed locally at the data centres of the participating cohort sites. Demographic, laboratory, and clinical data from each cohort are then pooled and analysed at the Data Coordinating Centre in Vancouver, BC. For this analysis, we focused exclusively on males, excluding men with missing or unknown data regarding MSM status. We also excluded individuals who did not have at least two viral load measurements within one year after initiating cART, as well as those with less than one year of follow-up. The date of administrative censoring for this analysis was 31 December 2012.

The human subjects activities of CANOC were approved by the Simon Fraser University Research Ethics Board, the University of British Columbia Research Ethics Board and the following local institutional review boards of the participating cohorts: Providence Health Care Research Institute Office of Research Services, The Ottawa Hospital Research Ethics Board, University Health Network (UHN) Research Ethics Board, Véritas Institutional Review Board (IRB), Biomedical C (BMC) Research Ethics Board of the McGill University Heath Centre (MUHC), University of Toronto HIV Research Ethics Board (HIV REB), and Women’s College Hospital Research Ethics Board.

Local cohort studies have obtained written consent except for the following: HAART Observational Medical Evaluation and Research (IRB approves the retrospective use of anonymous administrative data without requiring consent; an information sheet for participants is provided in lieu of a consent form); Ottawa Hospital Cohort (IRB approves the anonymous use of data retrospectively abstracted from clinical care databases without requiring consent); UHN (REB approves the anonymous use of data retrospectively abstracted from clinical care databases without requiring consent); MUHC (IRB approves the anonymous use of data retrospectively abstracted from clinical care databases without requiring consent; patients sign a general waiver on opening a medical chart at the hospital but no specific study related consent); Maple Leaf Medical Clinic (REB has approved the anonymous use of data retrospectively abstracted from clinical care databases without requiring consent); and Effective Anti-Retroviral Therapy cohort (REB approves the anonymous use of data retrospectively abstracted from clinical care databases without requiring consent; patients sign a general waiver on opening a medical chart at the hospital but no specific study related consent).

Further details on the collaborating cohorts and general CANOC structure are available [23].

### Outcomes

Viral suppression after cART initiation was defined as the time to the first of at least two consecutive plasma HIV RNA measurements below 50 copies/mL, at least 30 days apart in the first year of treatment. We defined suppression within one year to focus on more timely virologic control [25]. Viral rebound was only measured among MSM who achieved viral suppression within the first year of treatment. Rebound was defined as the time to the first of at least two consecutive VL measures above 200 copies/mL, at least 30 days apart.

### Statistical methods

Demographic and clinical characteristics at treatment initiation (baseline) were summarized using frequencies and proportions for categorical variables and medians and interquartile ranges (Q1–Q3) for continuous variables. Demographic and clinical characteristics of participants who achieved viral suppression within one year or did not achieve viral suppression within a year were compared using Chi-square tests for categorical variables and Wilcoxon Rank Sum tests for continuous variables.

Univariate accelerated failure time models with interval censoring were used to explore the association between covariates and each of the two outcomes. Covariates of interest included province of residence, race/ethnicity, age, baseline CD4 cell count, baseline viral load, history of injection drug use (IDU), baseline diagnosis of an AIDS-defining illness (ADI), era of cART initiation (i.e., 2000–2003, 2004–2007, 2008–2012), and third ARV class (i.e., NNRTI, boosted PI, unboosted PI, other). Calendar time of reaching viral suppression was also explored as a covariate in the rebound analysis because of its potential influence on subsequent rebound [26]. An exploratory model selection process based on Akaike Information Criterion (AIC), type III *p*-values, and a priori information was pursued to select variables for the multivariable accelerated failure time models examining time to viral suppression and rebound. The time origins were the first antiretroviral start date and the date of the first of two consecutive viral load measurements below 50 copies/mL and above 200 copies/mL, for the suppression and rebound models, respectively. The multivariable models were fitted to an exponential distribution. All analyses were performed using SAS statistical software, version 9.3 (SAS Institute, Cary, NC).

## Results

Baseline demographic and clinical characteristics of the study participants are listed in Table 1. Drawing on CANOC data from 2000 to 2011, 3,375 male participants were identified as MSM. 214 of these participants did not have at least two viral load measurements within one year after initiating cART, and another 303 participants had less than one year of follow-up. These participants were excluded from analysis, leaving a total of 2,858 men who met the eligibility criteria. By province, 30 % of participants were from BC, 37 % were from Ontario, and 34 % were from Quebec. The median follow-up time was 5.0 years (Q1–Q3: 3.0–8.1 years). There were 192 (7 %) participants lost to follow-up (defined as no contact for 18+ months), and 122 (4 %) died during follow-up. The median age of all participants was 40 years (Q1–Q3: 34–46 years). Among 1,441 participants with available ethnicity data, 1,320 (92 %) identified as White, 51 (4 %) as Black, and 70 (5 %) as Aboriginal. The median baseline CD4 count was 230 cells/mm^3^ (Q1–Q3: 130–321 cells/mm^3^) and the median baseline viral load was 4.96 log_10_ copies/mL (Q1–Q3 4.51–5.00 log_10_ copies/mL). Of the 2,744 participants with available hepatitis C (HCV) testing data, 331 (12 %) were seropositive. Additionally, 247 (9 %) participants had a history of IDU at the time of HIV diagnosis. At baseline, 459 (16 %) participants had been diagnosed with an ADI at or prior to cART initiation. The median rate of viral load testing was 4 tests per year (Q1–Q3: 3–5 tests).

**Table 1:** Characteristics of MSM study participants at enrolment into CANOC (*n* = 2858)

Characteristic	Total (%)^a^
Province	
British Columbia	854 (30)
Ontario	1045 (37)
Quebec	959 (34)
Age (years)	40 (34–46)
Ethnicity	
Caucasian	1320 (46)
Black	51 (2)
Aboriginal	70 (2)
Other	371 (13)
Unknown	1046 (37)
History of IDU	
No	2555 (89)
Yes	247 (9)
Unknown	56 (2)
Hepatitis C status	
No	2413 (84)
Yes	331 (12)
Unknown	114 (4)
Era of cART initiation	
2000–2003	729 (26)
2004–2007	931 (33)
2008–2012	1198 (42)
Number of viral load tests per year	
Less than 3	583 (20)
3–4	1590 (56)
5–6	360 (13)
More than 6	325 (11)
Initial 3rd ARV class	
NNRTI	1277 (45)
Unboosted PI	128 (4)
Boosted PI	1239 (43)
Other	214 (7)
Initial 3rd ARV	
Nevirapine	252 (9)
Efavirenz	1033 (36)
Lopinavir	424 (15)
Atazanavir	578 (20)
Other	571 (20)
NRTI combination	
Tenofovir/emtricitabine	1217 (43)
Zidovudine/lamivudine	594 (21)
Tenofovir/lamivudine	207 (7)
Abacavir/lamivudine	468 (16)
Stavudine/lamivudine	212 (7)
Other	160 (6)
AIDS-defining illness	
No	2284 (80)
Yes	459 (16)
Unknown	115 (4)
Viral load (log_10_ copies/mL)	4.96 (4.51–5.00)
CD4 count (cells/uL)	230 (130–321)

*ARV* antiretroviral, *IDU* injecting drug use, *NNRTI* nonnucleoside reverse transcriptase inhibitor, *PI* protease inhibitor

^a^Results are presented as *n* (%) with the exception of age, viral load, and CD4 count, for which median (Q1–Q3) is shown. Percentages may not equal 100 % as a result of rounding

Table 2 highlights characteristics associated with MSM achieving at least two consecutive plasma HIV RNA measurements below 50 copies/mL, at least 30 days apart in the first year of treatment. At noted here, 2,448 (86 %) MSM participants achieved viral suppression within 12 months of cART initiation. The median time to suppression was 5 months (Q1–Q3: 3–7 months). Of these 2,448 individuals, 295 (12 %) experienced a subsequent rebound above 200 HIV RNA copies/mL.

**Table 2:** Characteristics associated with MSM achieving viral suppression in the first year of treatment

Characteristic	Viral Suppression , n (%)		
	No (*n* = 410)	Yes (*n* = 2448)	*P*-value
Province			
British Columbia	140 (34)	714 (29)	0.011
Ontario	158 (39)	887 (36)	
Quebec	112 (27)	847 (35)	
Age (years)	38 (33–44)	40 (34–46)	0.002
Ethnicity			
Caucasian	216 (53)	1104 (45)	0.002
Black	8 (2)	43 (2)	
Aboriginal	17 (4)	53 (2)	
Other	40 (10)	331 (14)	
Unknown	129 (31)	917 (37)	
History of IDU			
No	336 (82)	2219 (91)	<0.001
Yes	60 (15)	187 (8)	
Unknown	14 (3)	42 (2)	
Hepatitis C status			
No	321 (78)	2092 (85)	0.001
Yes	68 (17)	263 (11)	
Unknown	21 (5)	93 (4)	
Era of cART initiation			
2000–2003	152 (37)	577 (24)	<0.001
2004–2007	127 (31)	804 (33)	
2008–2012	131 (32)	1067 (44)	
Number of viral load tests per year			
Less than 3	115 (28)	468 (19)	<0.001
3–4	188 (46)	1402 (57)	
5–6	48 (12)	312 (13)	
More than 6	59 (14)	266 (11)	
Initial 3rd ARV class			
NNRTI	140 (34)	1137 (46)	<0.001
Unboosted PI	39 (10)	89 (4)	
Boosted PI	194 (47)	1045 (43)	
Other	37 (9)	177 (7)	
Initial 3rd ARV			
Nevirapine	41 (10)	211 (9)	<0.001
Efavirenz	105 (26)	928 (38)	
Lopinavir	73 (18)	351 (14)	
Atazanavir	79 (19)	499 (20)	
Other	112 (27)	459 (19)	
NRTI combination			
Tenofovir/emtricitabine	134 (33)	1083 (44)	<0.001
Zidovudine/lamivudine	114 (28)	480 (20)	
Tenofovir/lamivudine	27 (7)	180 (7)	
Abacavir/lamivudine	58 (14)	410 (17)	
Stavudine/lamivudine	43 (10)	169 (7)	
Other	34 (8)	126 (5)	
AIDS-defining illness			
No	307 (75)	1977 (81)	0.006
Yes	88 (21)	371 (15)	
Unknown	15 (4)	100 (4)	
Viral load (log_10_ copies/mL)	5.00 (4.75–5.00)	4.93 (4.48–5.00)	<0.001
CD4 count (cells/uL)	190 (100–310)	231 (134–327)	0.001

In bivariate analysis, time to viral suppression was significantly associated with IDU history (*p* < 0.001), calendar year of cART initiation (*p* < 0.001), initial composition of ARV regimen (*p* < 0.001), baseline viral load (*p* < 0.001), baseline CD4 count (*p* = 0.001), HCV co-infection (*p* = 0.001), age (*p* = 0.002), ethnicity (*p* = 0.002), having an ADI at baseline (*p* = 0.006), and province of residence (*p* = 0.011).

Tables 3 and 4 present the univariate and multivariable results of the accelerated failure time suppression and rebound models. In adjusted multivariable analysis, MSM who experienced viral suppression within one year of treatment initiation were more likely to have initiated cART from 2004 to 2007 [adjusted hazard ratio (aHR) 1.26, 95 % confidence interval (CI) 1.11–1.42] and 2008–2012 [aHR 1.32, 95 % CI 1.17–1.48] compared with 2000–2003, and to be older [aHR 1.08 per decade, 95 % CI 1.03–1.13]. MSM who achieved suppressed viral loads were less likely to have an IDU history [aHR 0.71, 95 % CI 0.60–0.85], higher baseline viral load [aHR 0.73 per log10 copies/mL, 95 % CI 0.66–0.80], and an unboosted PI [aHR 0.60, 95 % CI 0.48–0.76] or boosted PI [aHR 0.81, 95 % CI 0.74–0.90] containing cART regimen (Table 3). The multivariable accelerated failure time model for viral rebound found that MSM who experienced a viral rebound were more likely to be younger [aHR 0.70 per decade, 95 % CI 0.62–0.80], to have an IDU history [aHR 2.52, 95 % CI 1.82–3.50], a higher CD4 cell count at baseline [aHR 1.13 per 100 cells/mm^3^, 95 % CI 1.05–1.22], and less likely to have initiated cART from 2004 to 2007 [aHR 0.69, 95 % CI 0.53–0.91] or 2008–2012 [aHR 0.43, 95 % CI 0.30–0.61], and to be living in Ontario [aHR 0.50, 95 % CI 0.38–0.67] or Quebec [aHR 0.51, 95 % CI 0.38–0.69] (Table 4).

**Table 3:** Accelerated failure time models of factors associated with time to viral suppression among MSM

	Unadjusted hazard ratio (95 % confidence interval)	*P*-value	Adjusted hazard ratio (95 % confidence interval)	*P*-value
Province				
British Columbia	1.00	0.004	1.00	0.126
Ontario	1.04 (0.93–1.15)		0.98 (0.88–1.10)	
Quebec	1.19 (1.07–1.32)		1.09 (0.98–1.22)	
Baseline age (per 10 year increment)	1.06 (1.02–1.11)	0.007	1.08 (1.03–1.13)	0.001
Ethnicity				
Caucasian	1.00	0.001		
Black	0.97 (0.68–1.39)			
Aboriginal	0.75 (0.55–1.01)			
Other	1.23 (1.07–1.40)			
Unknown	1.13 (1.03–1.25)			
History of IDU				
No	1.00	<0.001	1.00	<0.001
Yes	0.70 (0.60–0.83)		0.71 (0.60–0.85)	
Unknown	0.63 (0.44–0.89)		0.71 (0.50–1.02)	
Era of cART initiation				
2000–03	1.00	<0.001	1.00	<0.001
2004–07	1.24 (1.11–1.39)		1.26 (1.11–1.42)	
2008–12	1.42 (1.27–1.58)		1.32 (1.17–1.48)	
Initial 3rd ARV class				
NNRTI	1.00	<0.001	1.00	<0.001
Unboosted PI	0.53 (0.42–0.67)		0.60 (0.48–0.76)	
Boosted PI	0.82 (0.75–0.90)		0.81 (0.74–0.90)	
Other	0.81 (0.67–0.97)		0.83 (0.69–1.00)	
Baseline AIDS-defining illness				
No	1.00	0.011		
Yes	0.84 (0.74–0.94)			
Unknown	1.04 (0.84–1.29)			
Baseline viral load (per log10 copies/mL)	0.73 (0.66–0.80)	<0.001	0.73 (0.66–0.80)	<0.001
Baseline CD4 count (per 100 cells/mm^3^)	1.04 (1.02–1.07)	<0.001

**Table 4:** Accelerated failure time models of factors associated with time to viral rebound among MSM who suppressed

	Unadjusted hazard ratio (95 % confidence interval)	*P*-value	Adjusted hazard ratio (95 % confidence interval)	*P*-value
Province				
British Columbia	1.00	<0.001	1.00	<0.001
Ontario	0.54 (0.42–0.71)		0.50 (0.38–0.67)	
Quebec	0.48 (0.36–0.64)		0.51 (0.38–0.69)	
Baseline age (per 10 year increment)	0.75 (0.66–0.86)	<0.001	0.70 (0.62–0.80)	<0.001
Ethnicity				
Caucasian	1.00	0.008		
Black	1.35 (0.60–3.06)			
Aboriginal	1.67 (0.93–3.00)			
Other	0.98 (0.70–1.38)			
Unknown	0.67 (0.51–0.89)			
History of IDU				
No	1.00	<0.001	1.00	<0.001
Yes	2.67 (1.96–3.64)		2.52 (1.82–3.50)	
Unknown	2.02 (1.04–3.93)		2.18 (1.09–4.36)	
Era of cART initiation				
2000–03	1.00	<0.001	1.00	<0.001
2004–07	0.71 (0.56–0.92)		0.69 (0.53–0.91)	
2008–12	0.49 (0.35–0.69)		0.43 (0.30–0.61)	
Initial 3rd ARV class				
NNRTI	1.00	0.082	1.00	0.057
Unboosted PI	1.60 (0.99–2.59)		1.48 (0.90–2.42)	
Boosted PI	1.16 (0.91–1.49)		1.13 (0.86–1.48)	
Other	1.53 (1.01–2.31)		1.70 (1.12–2.59)	
Baseline AIDS-defining illness				
No	1.00			
Yes	0.99 (0.73–1.34)			
Unknown	1.00 (0.58–1.72)	0.999		
Baseline viral load (per log10 copies/mL)	1.11 (0.85–1.45)	0.454		
Baseline CD4 count (per 100 cells/mm^3^)	1.05 (0.97–1.13)	0.227	1.13 (1.05–1.22)	0.001
Time to suppression (months)	1.04 (0.99–1.09)	0.118	1.04 (0.99–1.09)	0.144

## Discussion

Close to 90 % of HIV-positive MSM receiving cART in CANOC achieved viral suppression within a median of 5 months of initiating treatment [27]. However, nearly one-in-eight HIV-positive MSM who achieved viral suppression on cART experienced viral rebound at some point following suppression during follow-up. This is the largest longitudinal, multi-provincial analysis of HIV treatment responses among MSM in Canada and demonstrates that MSM are close to UNAIDS’ proposed targets that 90 % of individuals on cART achieve viral suppression. This high rate suppression will ensure the long-term health of these men and limit new infections in this population.

The proportion of MSM achieving suppression in CANOC is similar to other studies from several international settings where the HIV burden is also predominantly concentrated among MSM. A large North American cohort study found that between 2001 and 2009, the cumulative incidence of 1-year viral suppression was 84 %, observing that MSM participants had a higher likelihood of achieving suppression compared with other male participants [25]. Between 2010 and 2012 in Australia, it was estimated that the proportion of HIV-positive MSM on cART with undetectable viral loads increased from 85 to 90 % [28]. Surveillance data from the United Kingdom (UK) suggests that 89 % of MSM who initiated cART in 2009 were virally suppressed by 2010 [29]. Unfortunately these international viral suppression estimates for large HIV-positive MSM populations do not provide follow-up regarding episodes of subsequent rebound. But, in a large UK-based cohort study of previously antiretroviral naïve PHA on cART, of which approximately half were MSM, 11 % experienced rebound [30], which is a similar proportion to MSM who experienced rebound in our analysis.

Consistent with previous research [31], our analysis found more recent calendar year of cART initiation to predict better treatment response within a large HIV-positive cohort. This may partially be explained by the simple fact that ARV regimens in more recent years have enhanced drug efficacy and reduced side effects, promoting more optimal treatment response. As part of our analysis, we also stratified antiretroviral regimens by era of cART initiation and found that in earlier calendar years, a significantly (*p* < 0.001) higher proportion of participants initiated treatment comprised of a Zidovudine and Lamivudine NRTI fixed combination (51, 23, and 1 % of participants from 2000 to 2003, 2004 to 2007, and 2009 to 2012, respectively). In contrast, the better-tolerated and more effective [32] Tenofovir and Emtricitabine fixed-dose regimen was the predominant baseline NRTI combination in later calendar years (0, 25, and 82 % of participants initiating cART from 2000 to 2003, 2004 to 2007, and 2009 to 2012, respectively). Furthermore, in terms of ARV drug class, regimens comprised of NNRTIs were more effective than either boosted or unboosted PI-based for the treatment of patients with no previous exposure to antiretroviral therapy. This is congruous with a meta-analysis examining ARV regimens among HIV-infected patients with limited or no previous exposure to antiretroviral therapy [33]. NNRTIs were also most prevalent in more recent calendar years, with 49, 33, and 51 % of participants initiating a regimen comprised of NNRTIs from 2000 to 2003, 2004 to 2007, and 2009 to 2012, respectively. More frequent rebound episodes, especially in British Columbia and in earlier calendar years, may also reflect physician-recommended structured treatment interruptions, which were more common during earlier cART eras and in British Columbia [34].

The low median baseline CD4 cell count among participants (230 cells/mm^3^) is concerning, although guidelines for the timing of cART initiation have changed significantly over the period of study. The median baseline CD4 cell count increased throughout the study timeline, likely in response to these updated guidelines. In 2000, the median baseline CD4 cell count among all participants was 183 cells/mm^3^, whereas in 2011, this measure was 358 cells/mm^3^. Although an improvement, other studies have demonstrated that PHA who initiate cART with CD4 cell measures below 350 cells/mm^3^ are at higher risk of AIDS and death than individuals who begin treatment at higher CD4 thresholds [35–37]. Recurrent viral load testing among PHA who started cART with lower CD4 counts is crucial for informing changes in ARV regimens that are necessary for preventing treatment failure and adverse health outcomes.

Our finding that higher baseline CD4 cell count was a significant predictor of viral rebound was surprising [38, 39]. It is possible that cART adherent MSM with high baseline CD4 cell counts retained comparatively better health throughout the study timeline, which may have influenced them to “take a break” from their medication [40, 41]. However, recent research set in the Canadian context has found no association between high CD4 counts (≥500 cells/mm^3^) and lower rates of adherence [42]. Going forward, it is important to examine this issue more closely. Recommendations for initiating cART immediately, regardless of CD4 cell count, emerged after our period of study, first in 2013 for select populations [10], and in 2015 for all PHA [37, 43]. As such, the proportion of PHA initiating cART at higher CD4 counts will increase in the future, providing an opportunity to obtain a better understanding of its predictive value regarding cART adherence.

This analysis discerned a few populations at risk of poorer virologic outcomes, including younger MSM and those with a history of injection drug use. Adverse treatment response was particularly evident in this latter group, who had 0.71 times the likelihood of achieving suppression, and over 2.5 times the chance of experiencing rebound. Their reduced probability of achieving and sustaining undetectable viral loads hinders UNAIDS suppression targets and has potential negative implications for future health outcomes, and on going HIV transmission. Younger MSM can face a wide array of challenges, including sexual identity issues, substance abuse, precarious employment, and housing instability [44]. Understandably, these conditions may complicate balancing a lifelong cART regimen and coming to terms with having a chronic and stigmatized disease. Health care providers with younger HIV-positive MSM patients should become more knowledgeable about local LGBT-focused hotlines, agencies, and media so that they can better connect them to these services, which may help improve HIV treatment self-efficacy and facilitate improved cART adherence [45]. Furthermore, HIV case management services should accommodate more flexible hours to promote continued engagement in care, as younger HIV-positive MSM are more likely to miss scheduled appointment times, and provide more flexible scheduling as this been has previously demonstrated to improve clinical care attendance and HIV treatment response among this population [46]. For improving treatment responses among MSM who also use injection drugs, more intensive case management and psychosocial support services have a strong record of improving retention in care among more vulnerable HIV-positive populations [47, 48]. For example, incorporating harm reduction services such as supervised injection facilities into HIV care settings and increasing the availability of methadone maintenance therapy may also lead to more consistent viral load suppression among MSM-IDU [49, 50].

### Strengths and limitations

Readers should be cautious when reviewing our findings even though our study was based on large group of MSM and 12-year follow-up period. Most notably, our study only included Canada’s three largest provinces, Ontario, Quebec and British Columbia, however, we did include most MSM as currently 85 % of PHAs in Canada live in these provinces [51]. The provincial differences in treatment response are likely explained by the fact that British Columbia includes the entire sample of CANOC-eligible individuals province-wide while data from Ontario and Québec included a selection of individuals from specialized HIV clinics (the majority being in urban centres). Men with missing or unknown MSM status (*n* = 2,073) were excluded from this analysis. Moreover, our results only pertain to MSM participants who had at least two viral load measurements within one year after initiating cART, as well as individuals with at least one year of follow-up. However, excluding this group from our analyses did not heavily bias our results, because male participants with unknown MSM status did not differ from the MSM sample significantly with regard to baseline CD4 cell count, achieving suppression, or experiencing rebound.

## Conclusions

Our results show that over 80 % of HIV-positive MSM with at least one year of follow-up achieved and maintained suppressed HIV viral levels. However, 12 % of MSM who achieved viral suppression went on to experience viral rebound within the study period. To better realize the UNAIDS 90 % suppression target, further strategies are required to optimize cART outcomes in MSM in Canada, specifically targeting younger MSM and those with a history of IDU.

## Appendix

The CANOC Collaborative Research Centre includes: CANOC Principal Investigator: Robert Hogg (British Columbia Centre for Excellence in HIV/AIDS, Simon Fraser University) Site Principal Investigators: Ann N. Burchell (Ontario HIV Treatment Network, University of Toronto, OHTN Cohort Study [OCS]), Curtis Cooper (University of Ottawa, OCS), Deborah Kelly (Memorial University of Newfoundland), Marina Klein (Montreal Chest Institute Immunodeficiency Service Cohort, McGill University), Mona Loutfy (University of Toronto, Maple Leaf Medical Clinic, OCS), Nima Machouf (Clinique Medicale l’Actuel, Université de Montréal), Julio Montaner (British Columbia Centre for Excellence in HIV/AIDS, University of British Columbia), Janet Raboud (University of Toronto, University Health Network, OCS), Chris Tsoukas (McGill University), Stephen Sanche (University of Saskatchewan), Alexander Wong (University of Saskatchewan) Co-Principal Investigators: Tony Antoniou (St. Michael’s Hospital, University of Toronto, Institute for Clinical Evaluative Sciences), Ahmed Bayoumi (St. Michael’s Hospital, University of Toronto), Mark Hull (British Columbia Centre for Excellence in HIV/AIDS), Bohdan Nosyk (British Columbia Centre for Excellence in HIV/AIDS, Simon Fraser University) Co-Investigators: Angela Cescon (Northern Ontario School of Medicine), Michelle Cotterchio (Cancer Care Ontario, University of Toronto), Charlie Goldsmith (Simon Fraser University), Silvia Guillemi (British Columbia Centre for Excellence in HIV/AIDS, University of British Columbia), P. Richard Harrigan (British Columbia Centre for Excellence in HIV/AIDS, University of British Columbia), Marianne Harris (St. Paul’s Hospital), Sean Hosein (CATIE), Sharon Johnston (Bruyère Research Institute, University of Ottawa), Claire Kendall (Bruyère Research Institute, University of Ottawa), Clare Liddy (Bruyère Research Institute, University of Ottawa), Viviane Lima (British Columbia Centre for Excellence in HIV/AIDS, University of British Columbia), David Marsh (Northern Ontario School of Medicine), David Moore (British Columbia Centre for Excellence in HIV/AIDS, University of British Columbia), Alexis Palmer (British Columbia Centre for Excellence in HIV/AIDS, Simon Fraser University), Sophie Patterson (British Columbia Centre for Excellence in HIV/AIDS, Simon Fraser University), Peter Phillips (British Columbia Centre for Excellence in HIV/AIDS, University of British Columbia), Anita Rachlis (University of Toronto, OCS), Sean B. Rourke (University of Toronto, OCS), Hasina Samji (British Columbia Centre for Excellence in HIV/AIDS), Marek Smieja (McMaster University), Benoit Trottier (Clinique Medicale l’Actuel, Université de Montréal), Mark Wainberg (McGill University, Lady Davis Institute for Medical Research), Sharon Walmsley (University Health Network, University of Toronto) Collaborators: Chris Archibald (Public Health Agency of Canada Centre for Communicable Diseases and Infection Control), Ken Clement (Canadian Aboriginal AIDS Network), Fred Crouzat (Maple Leaf Medical Clinic), Monique Doolittle-Romas (Canadian AIDS Society), Laurie Edmiston (Canadian Treatment Action Council), Sandra Gardner (OHTN, University of Toronto, OCS), Brian Huskins (Canadian Treatment Action Council), Jerry Lawless (University of Waterloo), Douglas Lee (University Health Network, University of Toronto, ICES), Renee Masching (Canadian Aboriginal AIDS Network), Stephen Tattle (Canadian Working Group on HIV & Rehabilitation), Alireza Zahirieh (Sunnybrook Health Sciences Centre) Analysts and Staff: Claire Allen (Regina General Hospital), Stryker Calvez (SHARE), Guillaume Colley (British Columbia Centre for Excellence in HIV/AIDS), Jason Chia (British Columbia Centre for Excellence in HIV/AIDS), Daniel Corsi (The Ottawa Hospital Immunodeficiency Clinic, Ottawa Hospital Research Institute), Louise Gilbert (Immune Deficiency Treatment Centre), Nada Gataric (British Columbia Centre for Excellence in HIV/AIDS), Alia Leslie (British Columbia Centre for Excellence in HIV/AIDS), Lucia Light (OHTN), David Mackie (The Ottawa Hospital), Costas Pexos (McGill University), Susan Shurgold (British Columbia Centre for Excellence in HIV/AIDS), Leah Szadkowski (University Health Network), Chrissi Galanakis (Clinique Médicale L’Actuel), Ina Sandler (Maple Leaf Medical Clinic), Benita Yip (British Columbia Centre for Excellence in HIV/AIDS), Jaime Younger (University Health Network), and Julia Zhu (British Columbia Centre for Excellence in HIV/AIDS).

## Abbreviations

ADI: AIDS-defining illness
AHR: Adjusted hazard ratio
AIC: Akaike Information Criterion
AIDS: Acquired immune deficiency syndrome
ARV: Antiretroviral
BC: British Columbia
CANOC: Canadian Observational Cohort
cART: Combination antiretroviral therapy
CI: Confidence interval
HCV: Hepatitis C
HIV: Human immunodeficiency virus
HR: Hazard ratio
IDU: Injection drug use
IRB: Institutional review board
LGBT: Lesbian, gay, bisexual and transgender
MSM: Men who have sex with men
MUHC: McGill University Health Centre
NNRTI: Nonnucleoside reverse transcriptase inhibitor
PHA: People living with HIV
PI: Protease inhibitor
REB: Research ethics board
RNA: Ribonucleic acid
UK: United Kingdom
UNAIDS: United Nations Programme on HIV/AIDS
